# Th2 to Th1 Transition Is Required for Induction of Skin Lesions in an Inducible and Recurrent Murine Model of Cutaneous Lupus–Like Inflammation

**DOI:** 10.3389/fimmu.2022.883375

**Published:** 2022-06-27

**Authors:** Nazgol-Sadat Haddadi, Purvi Mande, Tia Y. Brodeur, Kaiyuan Hao, Grace E. Ryan, Stephanie Moses, Sharon Subramanian, Xhuliana Picari, Khashayar Afshari, Ann Marshak-Rothstein, Jillian M. Richmond

**Affiliations:** ^1^ Department of Dermatology, University of Massachusetts Chan Medical School, Worcester, MA, United States; ^2^ Department of Medicine, Division of Rheumatology, University of Massachusetts Chan Medical School, Worcester, MA, United States

**Keywords:** cutaneous lupus erythematosus, CD4+ helper T cell, Lupus flare, CXCR6, Th1 & Th2

## Abstract

Cutaneous lupus erythematosus (CLE) is an autoimmune skin disease characterized by a strong IFN signature, normally associated with type I IFNs. However, increasing evidence points to an additional role for IFNγ, or at least a pathogenic T effector subset dependent on IFNγ, for disease progression. Nevertheless, Th2 effector subsets have also been implicated in CLE. We have now assessed the role of specific T cell subsets in the initiation and persistence of skin disease using a T cell-inducible murine model of CLE, dependent on KJ1-26 T cell recognition of an ovalbumin fusion protein. We found that only Th2-skewed cells, and not Th1-skewed cells, induced the development of skin lesions. However, we provide strong evidence that the Th2 disease-initiating cells convert to a more Th1-like functional phenotype *in vivo* by the time the skin lesions are apparent. This phenotype is maintained and potentiates over time, as T cells isolated from the skin, following a second induction of self-antigen, expressed more IFN-γ than T cells isolated at the time of the initial response. Transcriptional analysis identified additional changes in the KJ1-26 T cells at four weeks post injection, with higher expression levels of interferon stimulated genes (ISGs) including *CXCL9, IRF5, IFIH1*, and *MX1*. Further, injection of IFN-γ-/- T cells faied to induce skin disease in mice. We concluded that Th2 cells trigger skin lesion formation in CLE, and these cells switch to a Th1-like phenotype in the context of a TLR7-driven immune environment that is stable within the T cell memory compartment.

## Introduction

Cutaneous lupus erythematosus (CLE) is an autoimmune disease with a broad range of skin and mucosal tissue manifestations (1) that may or may not overlap with systemic lupus erythematosus (SLE). A high frequency of patients with SLE may develop skin lesions; however, not all CLE patients exhibit systemic disease or even progress to SLE (2). Additionally, CLE can be refractory to SLE treatments (3). Hence, pathogenic mechanisms need to be further explored to identify the unique and shared features of CLE and SLE, and to provide essential insights for treating both conditions.

A clear understanding of the immune parameters that contribute to CLE have been hampered by the lack of an appropriate experimental model, such that CLE has received less attention than SLE in terms of etiology and treatment. Well-characterized SLE animal models, including MRL/lpr, NZB/W, and BSXB mouse strains, have been used to study the development of skin lesions. However, in these strains, the onset of the cutaneous disease is variable, colony dependent, usually takes a long time (∼6 months) to develop and lacks a number of the critical features of human CLE (4). Recently, we have developed an inducible murine model of CLE that recapitulates human CLE by a number of criteria, including interface dermatitis, mucin deposition, lupus band reaction, erythema, scaling and hair loss (5). This model depends on the doxycycline induction of an OVA-peptide-containing pseudo-autoantigen (TGO), in combination with the adoptive transfer of activated OVA-specific DO11 T cells, and thereby allows us to explore the pathogenic activity of defined T cell subsets.

Early studies considered SLE an autoantibody/immune complex driven disease and focused on Th2 effector cells. However, the current literature points to a key role for skin-localized Th1 cells in lupus pathogenesis in both humans and mice (6–12). The serum levels of IFN-γ, TNF-α, and IL-12 are significantly higher in SLE patients than in healthy controls (11). The Th1-biased inflammation is most likely enhanced by type-I IFN secretion mainly from plasmacytoid dendritic cells (pDC) (13). By contrast, atopic dermatitis is normally associated with Th2 cells and psoriasis is driven by Th17 cells (14).

The TGO model was originally envisaged as a model of SLE since expression of the OVA pseudo-autoantigen is under the control of a reverse transactivator (rtTA) driven by an invariant chain promoter, and therefore likely to be expressed by all MHCII+ cells, not just MHCII+ cells in the skin. Based on the critical role of autoantibody/autoantigen immune complexes in lupus pathogenesis, all our initial studies used activated Th2-skewed T cells to initiate the disease process. The rapid onset of cutaneous lesions was unexpected, as was the finding that DO11 T cells isolated 4 weeks post transfer expressed a Th1 phenotype and produced IFNγ (5). The current study was therefore undertaken to better understand the role of Th2 vs Th1 cells in the development and recurrence of CLE. Unexpectedly, we found that an initial injection of Th1 cells failed to trigger cutaneous lesions, even though the injected DO11 T effector cells were present in the spleen, LN and to some extent the skin. Together, our data point to the distinct ability of Th2 cells to migrate to the skin and the unexpected plasticity of these Th2 cells to acquire a Th1-like phenotype when exposed to an immune environment perturbed by a TLR7-driven type I IFN response.

## Results

### Analysis of *In Vitro* Skewed DO11 T Cells Confirms Cytokine and Gene Expression Associated with Th1 and Th2 Differentiation

**Figure 1:**
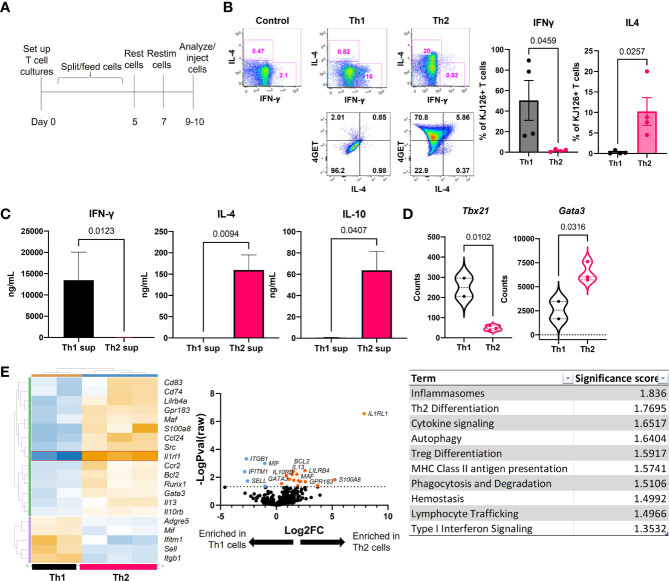
Generation and functional phenotype of activated DO11 T cells. **(A)** Timeline for the generation of activated Th1 and Th2 subsets. **(B)** Cytokine production by the injected Th1 and Th2 cells shown by flow cytometric analysis of IL-4 vs IFNγ (top) and GFP vs IL-4 (bottom). **(C)** Culture supernatants from the restimulated Th1 and Th2 cells determined by ELISA (n=5-6 Th1 and 9-11 Th2). **(D)** NanoString analysis of master regulator transcription factors expressed by *in vitro* activated T cells. **(E)** NanoString heatmap, volcano plot and Gene Set Analysis (GSA) of restimulated Th1 and Th2 cells analyzed by Rosalind software (n=2 Th1 and n=3 Th2 RNA samples pooled from 2 separate experiments; two-tailed student’s t tests significant as indicated).

Ii-TGO mice were generated by intercrossing mice that express an invariant chain promoter-driven reverse transactivator (Ii-rtTA) transgene with mice that express a Tet-regulated ovalbumin fusion protein (TRE-TGO). Upon Dox administration, these mice express an OVA fusion protein that incorporates the transferrin receptor transmembrane domain to facilitate efficient trafficking to endocytic compartments. To compare the pathogenic potential of distinct T cell subsets, OVA-specific DO11 T cells were activated *in vitro* with OVA-peptide and APCs under Th1 or Th2 skewing conditions, expanded in the presence of IL-2, and then restimulated 2-3 days prior to i.v. injection (**Figure 1A**). In some studies, we used T cells derived from DO11 mice that had been intercrossed with the IL-4 reporter line, 4get (15). We confirmed the functional phenotype of the Th1 and Th2 cells at the time of injection by flow cytometry. Only the Th1 cells expressed IFNγ, and only the Th2 cells expressed IL-4. In addition, the majority of the OTII 4get Th2 cells expressed GFP while few if any of the OTII 4get Th1 cells were GFP+, confirming their commitment to the Th1 lineage (**Figure 1B**). Cytokine concentrations in culture fluids collected after the *in vitro* restimulation were determined by ELISA, and the results confirmed the expected phenotype; Th1 supernatants contained high levels of IFNγ and the Th2 supernatants contained IL-4 and IL-10 (**Figure 1C**).

In addition, RNA extracted from the restimulated T cells was analyzed by NanoString™ Mouse Immunology code set. *Tbx21* (Tbet) was enriched in Th1 cells and *Gata3* was enriched in Th2 cells (**Figure 1D**). Additional genes were differentially upregulated in the two populations, including increased *S100a8, Il13, Il10rb*, and *Runx1* in Th2 cells and increased *Mif, Itgb1*, *Ifitm1* and *Sell* in Th1 cells (heatmap) (**Figure 1E**). The highest upregulated differentially expressed gene (DEG) in Th2 cells was *Il1rl1* (volcano plot). Gene set analysis (GSA) revealed Th2 differentiation, lymphocyte trafficking and type I interferon signaling terms were different between Th1 and Th2 cells using a cutoff significance score of 1.3.

### Injection of Th2 Cells, and Not Th1 Cells, Induces Skin Disease in Lupus-Prone Mice

**Figure 2:**
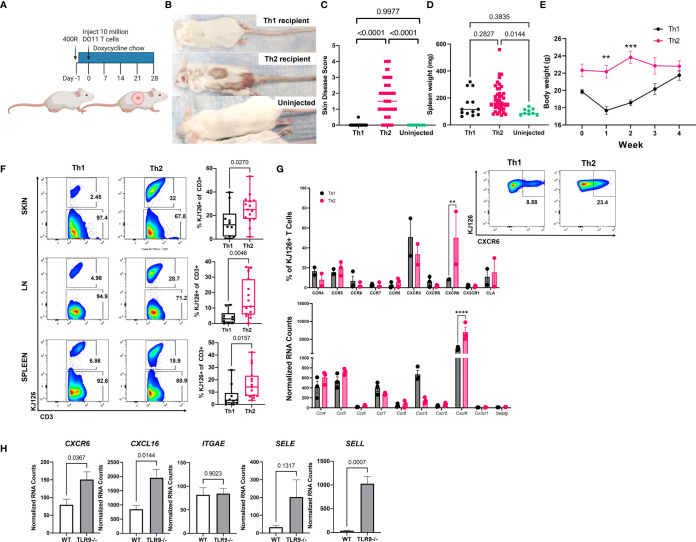
Injection of Th2, but not Th1, induces lupus-like skin lesions in mice. **(A)** Timeline for the initial induction of skin lesions created with BioRender.com. **(B)** Representative images of mice injected with Th1 or Th2 cells, compared to uninjected control. **(C)** Skin disease scores, **(D)** spleen weights and **(E)** body weights of mice (n=15 Th1, 45 Th2 and 9 uninjected mice pooled from 4 separate experiments, one and two way ANOVAs significant as indicated.) **(F)** T cell engraftment assessed by flow cytometry analysis of cell suspensions recovered from skin, lymph node (LN) and spleen tissue (n=10 Th1 and n=15 Th2 mice pooled from 2 separate experiments; two-tailed students t tests significant as indicated). **(G)**. Assessment of chemokine receptor expression by RNA (top) and flow cytometry (bottom) of *in vitro* activated Th1 and Th2 cells. **(H)** Reanalysis of gene expression in total skin of TLR9-/- versus WT control mice from Mande et al., (5) to assess ligand expression. **p<0.01, ***p<0.001 and ****p<0.0001.

We reported previously that sublethally irradiated (400R) IiTGO mice, provided with Dox chow and injected with activated Th2 cells, developed lupus-like skin lesions (5). To determine whether Th1 cells could induce CLE as efficiently as Th2 cells, TLR9^-/-^ Ii-TGO recipients were sublethally irradiated (400R) and provided with Dox chow 6-18 hrs prior to i.v. injection of Th1 or Th2 DO11 T cells (**Figure 2A**). Skin lesions developed in the Th2 injected mice 3-4 weeks post transfer, but not in the mice injected with Th1 cells, as shown in images of representative mice (**Figure 2B** **)** and compiled skin score data from 4 experiments **(** **Figure 2C**). The absence of skin lesions in the Th1-injected mice was not due to the failure of KJ1-26 cells to survive or engraft the recipients, as indicated by splenomegaly (**Figure 2D**) and the initial weight loss in the Th1-injected mice (**Figure 2E**). To further compare the extent of engraftment of the injected DO11 Th1 and Th2 T cells, single cell suspensions obtained from the skin, LN and spleen of the injected mice were analyzed by flow cytometry using the KJ1-26 anti-clonotypic antibody. We found more KJ1-26+ cells in Th2 injected mice in all 3 tissues (**Figure 2F**).

**Figure 3:**
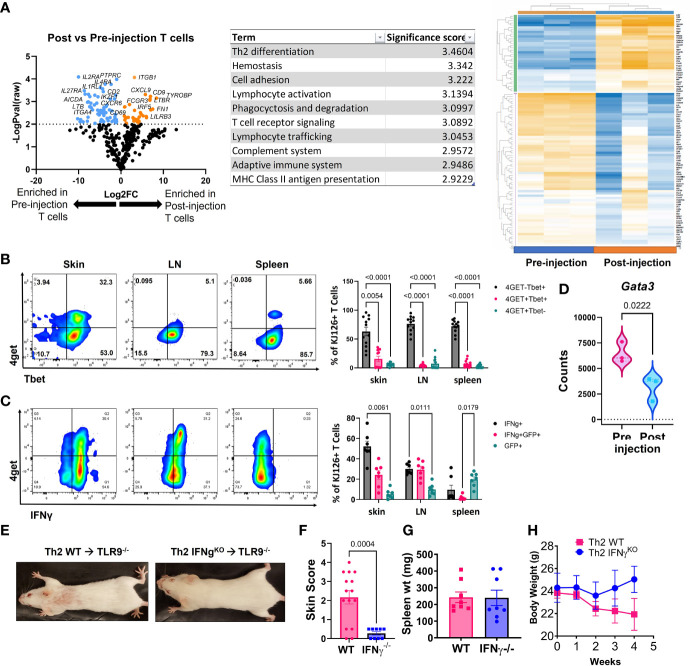
Analysis of post-injection T cells reveals a Th1-shift, and IFNγ is required for development of skin lesions. **(A)** NanoString analysis of post vs pre-injection T cells including volcano plot, GSA and heatmap generated with Rosalind software (n=3 pre-injection and n=3 post-injection enriched T cells pooled from 2 separate experiments). **(B)** Flow cytometry analysis of 4get reporter and Tbet expression in skin, lymph node (LN) and spleen of mice exhibits a Th1 switch post-injection (n=7 mice pooled from 2 separate experiments; two-way ANOVA with Tukey’s post-tests significant as indicated). **(C)** Flow cytometry analysis of 4get reporter and IFNγ expression in skin, lymph node (LN) and spleen of mice exhibits a Th1 switch post-injection (n=12 mice pooled from 2 separate experiments; two-way ANOVA with Tukey’s post-tests significant as indicated). **(D)** Assessment of *Gata3* RNA expression in pre- vs post-injection Th2 cells (n=3 pre-injection and n=3 post-injection T cells pooled from 2 separate experiments; one-tailed student’s t test significant as indicated). **(E)** Representative images of mice injected with Th2 skewed WT or IFNγ-/- DO11 cells. **(F)** Skin disease scores, **(G)** spleen weights and **(H)** body weights of mice (n=15 WT and 9 IFNγ-/- DO11 recipient mice pooled from 2 separate experiments, student’s t tests significant as indicated).

Th1 and Th2 cells are known to express distinct sets of chemokine receptors and Th1 and Th2 cells use different ligands and chemokine receptors to enter the skin (14). In addition to their role in cell migration, chemokine receptors impart functional capacity on T cells. Therefore, we examined DEGs between pre- and post-injection T cells, as well as Th1 and Th2 skewed cells, to better understand factors that might be contributing to T cell function in our model. CXCR6 was significantly higher in Th2 vs. Th1 cells by both NanoString array and flow cytometry (**Figure 2G**). However, there were no significant differences in the expression of other chemokine receptors, based on both Nanostring and flow panels, that have been reported to distinguish Th2 cells from Th1 cells (e.g., CXCR3, CCR4, CCR5, or CCR8) (**Figure 3G**), even though the cells were clearly skewed to the Th2 subset, based on cytokine production and expression of the 4get reporter (**Figure 1**).

To confirm the presence of the ligands for CXCR6 and CLA in CLE mouse skin, we queried our NanoString dataset which compared RNA isolated from the skin of Th2 injected TLR9^-/-^ Ii-TGO mice (skin score 3-4) to the skin of TLR9+/+ IiTGO mice (no disease) (5). We found significant increases in *CXCL16* (ligand for CXCR6) and *SELL* (ligand for CLA) but not *SELE* (another ligand for CLA) in TLR9-/- versus WT skin (**Figure 2H**). Taken together, these data suggest that Th2 skewing promotes skin infiltration by patterning expression of skin-homing molecules, thereby allowing the Th2 cells to follow CXCL16-CXCR6 chemokine and CLA-L-Selectin integrin signals to impart functional capacities in skin.

### Antigen-Specific Th2 Cells Switch to Th1-Like Cells *In Vivo*

Next, we compared the injected KJ1-26 Th2 cells to KJ1-26 T cells isolated from lesional skin 4-5 weeks post injection to identify changes in gene expression that developed during the post-injection time frame. Antigen-specific T cells were enriched from the skin using KJ1-26 magnetic beads for positive selection. NanoString analysis of RNA isolated from these cells revealed 150 differentially expressed genes (DEGs) between injected and 4 wk post-injection KJ1-26+ T cells. T cells isolated from lesional skin showed the upregulation of interferon-stimulated genes (ISGs), including *IRF5*, *IFIH1*, and *MX1* (**Figure 3A**). The data also showed decreased expression of Th2-related genes including *IL4RA*. These data, combined with the observation that the Th2 upregulated other ISGs led us to further examine the cytokine profile of post-injection DO11 4get T cells.

We tracked 4get expression in both *in vitro* and *ex vivo* from T cells, in addition to staining for IFNγ. By 4 weeks post Th2 cell injection a high proportion of KJ1-26+ T cells in the skin and skin-draining LN (sdLN) exhibited a Th1-like phenotype, as defined by Tbet and IFN-γ expression (**Figures 3B, C** **)**. Intriguingly, ~75% of the T cells in the skin were Tbet+/IFNg+ and of these, 25% also were also GFP^+^. Lower GFP detection in combination with Tbet staining may be due to the transcription factor fixation/nuclear permeabilization protocol, which is more harsh than the reagents used for intracellular cytokine staining. *Gata3*, a master regulator of Th2-associated gene expression, was also reduced in the post-injection T cells expression (**Figure 3D**). Together, these data indicate that the injected KJ1-26+ T cells switch from a Th2 to a Th1-like subset and acquire the capacity to produce IFNγ.

### DO11 IFN-γ Production Is Required for Skin Disease

To understand the significance of IFN-γ to development of skin disease, we skewed WT or IFN-γ^-/-^ DO11 T cells towards a Th2 phenotype and injected them into host mice. IFN-γ^-/-^ DO11 failed to induce skin lesions in mice (**Figures 3E, F** **)**, despite inducing splenomegaly and an initial drop in body weight (**Figures 3G, H** **)**. These data indicate that the switch towards a Th1 phenotype is required for the development of skin disease in this model.

### DO11 Memory T Cells Maintain Their Th1 Phenotype Following Reinduction of Skin Disease

**Figure 4:**
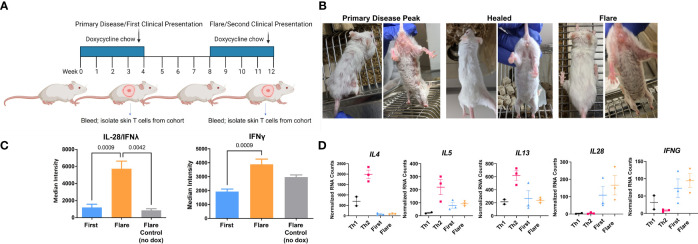
*In vivo* acquired Th1 phenotype is maintained by memory T cells. **(A)** CLE flare model diagram created with BioRender.com. **(B)** Sample clinical photographs of initial induction and reinduction of skin lesions. **(C)** RayBiotech array analysis of serum from mice at the time of the initial occurrence of skin lesions and the reinduction of skin lesions. **(D)** Comparison of *in vitro* activated T cells and KJ-126+ T cells recovered from the initial and reinduced skin lesion by NanoString gene expression analysis (n=2-3 cultured cell batches and 3 mice per group pooled from 2 independent experiments).

CLE is cyclical, with relapsing and remitting flares of cutaneous lesions. To model disease flares, mice that had developed skin disease were taken off Dox chow to allow the skin lesions to heal. Dox chow was then readministered to these mice 4 weeks later to reinduce the model autoantigen (**Figure 4A**). Within two weeks, skin disease recurred (**Figure 4B**) without the injection of additional DO11 T cells. Sera collected during the initial clinical presentation and at the time of flare were assayed by protein array to determine whether cytokine titers increased in the mice with flares. We found increases in IL28 (IFNλ) and IFN-γ in flares versus initial clinical presentation (**Figure 4C**). Cytokine titers in the sera of flare control mice, which did not receive the second course of Dox chow, were comparable to those detected during the initial response.

To determine whether these cytokines could be T cell derived, RNA isolated from the cultured Th1 and Th2 cells was compared to T cells isolated from the skin during the initial and flare responses. Gene transcription was assessed with the NanoString probe set as in **Figure 3**. We found that T cells isolated from the skin showed minimal expression of *IL4, IL5* and *IL13*, but did express *IL28* and *IFNγ*, both of which trended higher at time of flare (**Figure 4D** **)** Taken together, these data indicated that the Th1-like phenotype, established during the primary response, persisted in the T cell memory compartment.

## Discussion

In the current study we have explored the role of specific T cell subsets in an SLE-like model of inflammatory skin disease. We found that the injection of *in vitro* skewed Th2 cells induced skin lesions under conditions where *in vitro* skewed Th1 cells did not. Nevertheless, antigen-specific T cells isolated from lesional skin 4 weeks post the initial induction of disease displayed a Th1-like and not Th2-like phenotype, and the acquired Th1 phenotype persisted in memory T cells even when the autoantigen levels decreased upon withdrawal of doxycycline chow.

The injected Th2 cells preferentially expressed CXCR6, consistent with reports that CXCR6 mediates skin homing. The ligand for CXCR6, CXCL16, was recently reported to be elevated in juvenile SLE patients, and was strongly associated with alopecia, malar rash, and nephritis (16). CXCL16 is produced by keratinocytes (17, 18), and induced by TLR7 ligation (19). TLR7 plays a key role in the current CLE mouse model (5) as well as in human CLE [reviewed in (20)]. Further, CXCL16 is constitutively expressed by keratinocytes and is upregulated by UV light, a known trigger of CLE (18). Recent studies have also identified CXCR6 on skin effector T cells in the context of melanoma (21) and on skin resident memory T cells (Trm) in melanoma-associated vitiligo (22).

Th2 cells were long believed to be stably committed effector cells that were resistant to Th1 skewing conditions. However, there is precedent for Th2 cells to retain a degree of plasticity that enables them to adapt to changes in their microenvironment [reviewed in (23)] Examples include the role of increased type I IFN signaling following lymphocytic choriomeningitis (LCMV) viral challenge (24, 25). The Th2 cells used to induce disease in the current study produced IL-4 and failed to produce IFNγ, but they were only cultured with skewing antibodies and cytokines during the initial culture period and may not have been fully committed to the Th2 lineage (26). We hypothesize that in the immune microenvironment of CLE mice, TLR7-driven production of type I IFNs by pDCs, keratinocytes or other innate immune effector populations may skew the adoptively transferred Th2 cells towards a Th1 phenotype. Despite their inability to produce either IL4 protein or IL4 transcripts, a significant number of 4-GET KJ1-26+ cells in the CLE mice still expressed GFP, perhaps reflecting an active IL-4 promoter. Nevertheless, expression of *Gata3*, the master regulator of Th2 differentiation was significantly reduced in cells isolated from the lesional skin at weeks when compared to cultured Th2s. Hence, future studies will explore the histone methylation status of the Th1 or Th2 associated promoters during different stages of CLE pathogenesis.

Nevertheless, we cannot rule out the possibility that non-Th2 cells present in the initial innoculum further developed *in vivo* into disease inducing Th1 effector cells. We believe this is unlikely, since we have never found that the injection of Th1 cells led to the development of skin disease. It is also possible that activated Th2 cells have a selective survival advantage *in vivo* since they do not express the high levels of FasL found on Th1 cells, but in preliminary studies, DO11 Fas-deficient (lpr/lpr) Th1 cells still failed to induce skin disease.

Our findings showed higher levels of other serum cytokines including IL-12p70, IL-28 (also known as IFN-λ2/3), and TNF-α during CLE flare compared to the initial response. IFNλs have type I IFN-like activity and act primarily on epithelial cells. There are reports of high IFNλ and the IFNλ receptor in keratinocytes of CLE lesional skin (27). Elevated blood levels of IFNλ3, as well as increased *IFNL2* and *IFNL3* mRNA have been detected in blood CD4+ T cells of lupus-prone mice and patients (28, 29). TNF family members are elevated in lupus patients (30) and are shed at higher rates preceding flares (31). Further, elevated serum levels of IL-4, IL-5, IL-6, and IFN-γ precede autoantibody positivity in systemic lupus patients (32). Taken together, these studies of serum cytokines mirror what we observed in our flare model and support the idea that a plastic T cell pool may promote clinical manifestations in human patients.

In conclusion, our findings highlight the role of Th2 in the initiation of skin lupus in mice. We hypothesize that differences in chemokine receptors and ligands expressed on Th2 vs. Th1 cells enable Th2 cells to enter the skin and establish disease. *In vivo*, Th2 cells acquire an IFN-γ^+^ phenotype associated with the establishment and maintenance of skin disease. We also found that the IFN-γ producing function of Th cells is potentiated during the flare. One interesting implication of the current study is that Th2 cells, responding to foreign allergens in the skin, may on occasion recognize other self or foreign epitopes, and in the context of an ongoing inflammatory response, trigger the onset of SLE. The ability to readily induce flares in this model also point to persistent autoreactive T resident memory cells as potential therapeutic targets.

## Materials and Methods

### Mice

All mice were housed in pathogen-free facilities at UMMS, and procedures were approved under protocol #2096 by the UMMS Institutional Animal Care and Use Committee and in accordance with the National Institutes of Health (NIH) Guide for the Care and Use of Laboratory Animals. Mice used for these studies were on the Balb/c background. Age and sex-matched mice were used, and both male and female mice of all strains were tested to avoid gender bias. Replicate experiments were performed two to five times.

Recipient TLR9KO Ii-TGO and WT Ii-TGO controls were generated as previously described (5). BALB/c DO11 mice (C.Cg-Tg [DO11.10]10 Dlo/J; Jackson Laboratory stock no. 003303) or Rag-/- DO11 mice (C.Cg-Rag1tm1Mom Tg(DO11.10)10Dlo/J; stock no. 030666) bred to IL-4/GFP-enhanced transcript (4get) mice (C.129-Il4tm1Lky/J; stock no. 004190) were used as T cell donors.

### T Cell Skewing

Magnetic bead-purified DO11 CD4+ T cells (BD IMag magnetic particles) were activated using OVA peptide–pulsed (323-339, *In vivo*gen) irradiated spleen cells (as source of APCs) as described previously (33). Th1 cells were cultured with recombinant mouse IFNγ (10ng/mL) and anti-IL4 antibody (10ug/mL); Th2 cells were cultured with recombinant mouse IL-4 (10ng/mL) and anti-IFNγ (XMG2.1 10ug/mL) and anti-IL12p40 (10ug/mL). All cells (including unskewed Th0) received recombinant mouse IL-2 from J2 supernatant to promote survival and expansion. Cells were split on day 2, fed IL-2 on day 3, and split again on day 4. By day 7, the cells had rested and were re-activated, but not re-skewed, with another batch of OVA-pulsed splenocytes. Cells were harvested on day 10 at the peak of activation post-restim.

### RayBiotech Array

Supernatants from restimulated T cells and/or serum from CLE mice were assayed in the RayBiotech Th1/Th2/Th17 mouse Quantibody array per the manufacturer’s protocol. Slides were shipped for scanning array service and data were analyzed by taking the median fluorescence intensity minus the background fluorescence from blank control wells. Data are deposited on GEO Database under accession # GSE186095.

### NanoString Analysis

RNA was extracted from polarized cultured T cells, and from post-injection CD3 column-enriched (Miltenyi biotech) T cells from CLE mouse skin using Qiagen RNEasy mini kits. RNA was hybridized for ~18h (BioRad CFX thermocycler) and assayed in the NanoString mouse Immunology panel per the manufacturer’s instructions. Data were analyzed with Rosalind software using NanoString partner analysis. Data are deposited on GEO Database under accession # GSE185355.

### CLE Induction

10^7 activated and skewed T cells were injected i.v. into sublethally irradiated (4 Gy) age- and sex-matched TLR9WT or TLR9KO Ii-TGO recipient mice. To induce expression of the TGO transgene in the MHCII cells, mice were fed with 200 mg/kg of Dox chow (Bio-Serv). For CLE flares, mice were kept on chow for 4-5 weeks, allowed to heal for 4 weeks, then Dox chow was reintroduced.

### Flow Cytometry

Single-cell suspensions obtained from spleen, sdLNs, and skin were analyzed by flow cytometry using fluorochrome-conjugated mAbs listed in **Table S1**. Zombie Aqua or Zombie NIR (Biolegend) was used to distinguish live and dead cells. Intracellular staining was carried out on cells incubated with Brefeldin A (Biolegend) in all tissue digestion and FACS staining buffers, approximately for 4 hours. Cells were permeabilized and fixed with transcription factor staining buffer (Invitrogen) or Cytofix/Cytoperm (BD Biosciences) and subsequently incubated with fluorochrome-conjugated mAb to mouse IFN-γ (clone XMG1.2, eBioscience), IL4 (clone 11B11, Biolegend), Tbet (clone 4B10, Biolegend), or GATA3 (clone TWAJ, eBioscience). Flow cytometric analysis was carried out using a Cytek Aurora, and analysis was conducted with FlowJo software 9.7.6 (TreeStar).

### Cell Isolation From Skin

Cells were isolated from the skin as described previously (5). Briefly, shaved dorsal skin was harvested, minced, and digested for 45 minutes at 37°C with 2.0 mg/ml collagenase XI from Clostridium histolyticum (Sigma-Aldrich), 0.5 mg/ml hyaluronidase from bovine testes (Sigma-Aldrich), and 0.1 mg/ml DNAse (Sigma-Aldrich). Single cells were washed with 10% cRPMI, filtered through a 100 μm filter, and stained for flow cytometry staining as described above. For samples to be used for ICS, Brefeldin A was added to the digestion buffer and surface stain cocktail. For enrichment of antigen-specific T cells from skin, we used PE-conjugated KJ1-26 antibody and a PE positive selection kit (Miltenyi biotech).

### Statistics

Statistical analyses were performed using Prism software version 7.0 (GraphPad). Experiments are reported as mean ± SEM. Data were analyzed using a 2-tailed Student’s t test for comparison between 2 data sets. Multiple comparisons were analyzed by 1-way ANOVA and 2-way ANOVA, followed by Tukey’s multiple-comparison *post hoc* test. Differences were considered significant at a P value of less than 0.05.

## Data Availability Statement

The datasets presented in this study can be found in online repositories. The names of the repository/repositories and accession number(s) can be found below: https://www.ncbi.nlm.nih.gov/geo/, SuperSeries #GSE186096, containing accession # GSE186095 and GSE185355.

## Ethics Statement

The animal study was reviewed and approved by University of Massachusetts Chan Medical School Institutional Animal Care and Use Committee.

## Author Contributions

Conceptualization: AM-R and JR; Methodology: AM-R and JR; Software Programming: N/A; Validation/Verification: GR, N-SH, and KA; Formal analysis: N-SH, PM, GR, and JR; Investigation: N-SH, PM, TB, KP, SS, SM, KH, KA, AM-R, and JR; Resources: JR and AM-R; Data Curation: N-SH, PM, and JR; Writing - Original Draft: N-SH and JR; Writing - Review and Editing: All authors; Visualization: N-SH, PM, KH, TB, JR, and AM-R; Supervision: JR and AM-R; Project administration: JR and AM-R; Funding acquisition: JR and AM-R. All authors contributed to the article and approved the submitted version.

## Funding

Supported by NIH grants 1R21AI136253 - 01A1, 1R21 AI145097-02 (to AM-R), a Women’s Health Career Development Award from the Dermatology Foundation, a Target Identification in Lupus Award from the Lupus Research Alliance, and a Pilot Program Project grant from the UMass Center for Clinical and Translational Science, made possible through NIH grant # UL1-TR001453 (to JR). Flow cytometry and confocal microscopy equipment used for this study is maintained by the UMass Chan Flow Cytometry Core Facility and Morphology Core Facility.

## Conflict of Interest

Author PM is employed by Q32 Bio Inc. JR is an inventor on patent application #15/851,651, “Anti-human CXCR3 antibodies for the Treatment of Vitiligo” which covers targeting CXCR3 for the treatment of vitiligo; and on patent #62489191, “Diagnosis and Treatment of Vitiligo” which covers targeting IL-15 and Trm for the treatment of vitiligo.

The remaining authors declare that the research was conducted in the absence of any commercial or financial relationships that could be construed as a potential conflict of interest.

## Publisher’s Note

All claims expressed in this article are solely those of the authors and do not necessarily represent those of their affiliated organizations, or those of the publisher, the editors and the reviewers. Any product that may be evaluated in this article, or claim that may be made by its manufacturer, is not guaranteed or endorsed by the publisher.
